# The role of accessory proteins and co‐factors in regulation of melanocortin‐4 receptor signalling: An update

**DOI:** 10.1111/jne.70160

**Published:** 2026-03-16

**Authors:** Aqfan Jamaluddin, Alyce McClellan, Eleanor Raffan, Caroline M. Gorvin

**Affiliations:** ^1^ Department of Metabolism and Systems Science, School of Medical Sciences, College of Medicine and Health University of Birmingham Birmingham UK; ^2^ Centre of Membrane Proteins and Receptors (COMPARE) Universities of Birmingham and Nottingham Birmingham UK; ^3^ Department of Structural and Molecular Biology University College London London UK; ^4^ Department of Physiology, Development and Neuroscience University of Cambridge Cambridge UK; ^5^ Metabolic Research Laboratories Institute of Metabolic Science, University of Cambridge Cambridge UK

**Keywords:** appetite, endocytosis, G protein‐coupled receptor, metabolism

## Abstract

The melanocortin‐4 receptor (MC4R) is a G protein‐coupled receptor with an essential role in appetite suppression and energy homeostasis. Genetic mutations in the receptor and components of its signalling pathway that cause obesity in humans, dogs and rodent models have revealed important insights into how the receptor signals and what regulates its cell surface expression. Structural studies have identified calcium as a critical cofactor for agonist binding and receptor function, while several transmembrane proteins have been shown to modulate MC4R activity. Here, we describe recent developments in our understanding of how accessory proteins and cofactors, identified using genomic approaches and screens for protein interaction, modify MC4R trafficking and signalling. We discuss how signalling by G_s_ and G_q/11_ pathways may have differential effects on food intake, weight gain and cardiovascular function. We also summarise recent studies of MC4R expression at primary cilia, receptor oligomerisation, newly identified proteins that regulate MC4R cell surface expression, and briefly discuss novel endogenous agonists.

## 1 INTRODUCTION

The melanocortin‐4 receptor (MC4R) is a class A G protein‐coupled receptor (GPCR) primarily expressed at the paraventricular nucleus (PVN) of the hypothalamus where it occupies an essential role in the melanocortin‐leptin pathway that regulates food intake and energy homeostasis. MC4R is unusual as it has both endogenous agonists, the proopiomelanocortin (POMC)‐derived peptides alpha‐melanocyte‐stimulating hormone (α‐MSH) and β‐MSH that suppress appetite, and an endogenous antagonist, the agouti‐related peptide (AgRP), which promotes food intake. POMC and AgRP are derived from different neuronal populations of the hypothalamic arcuate nucleus. Depletion of the *Mc4r* gene from mice causes hyperphagic obesity and genetic variants that inactivate MC4R activity cause severe early‐onset obesity in humans with hyperphagia, hyperinsulinemia, dyslipidaemia, abnormal linear growth and higher risk of cardiovascular complications.^1^ Studies of some of these MC4R mutant proteins have revealed insights into how the receptor activates and signals, and investigation of human populations with overweight and/or obesity has identified proteins that modulate MC4R signalling and/or trafficking.^2^ The development of synthetic MC4R‐selective agonists such as setmelanotide that reduce weight and hunger in individuals with mutations in the MC4R pathway have shown that pharmacologically targeting the MC4R pathway is possible.^3^ This review will summarise new insights into the way in which MC4R signals, focussing on how accessory proteins including other membrane proteins, inorganic cofactors and trafficking proteins modulate MC4R function. Additionally, we will briefly introduce additional endogenous agonists that may directly bind to the receptor.

## 2 CURRENT UNDERSTANDING OF MC4R SIGNALLING

**FIGURE 1:**
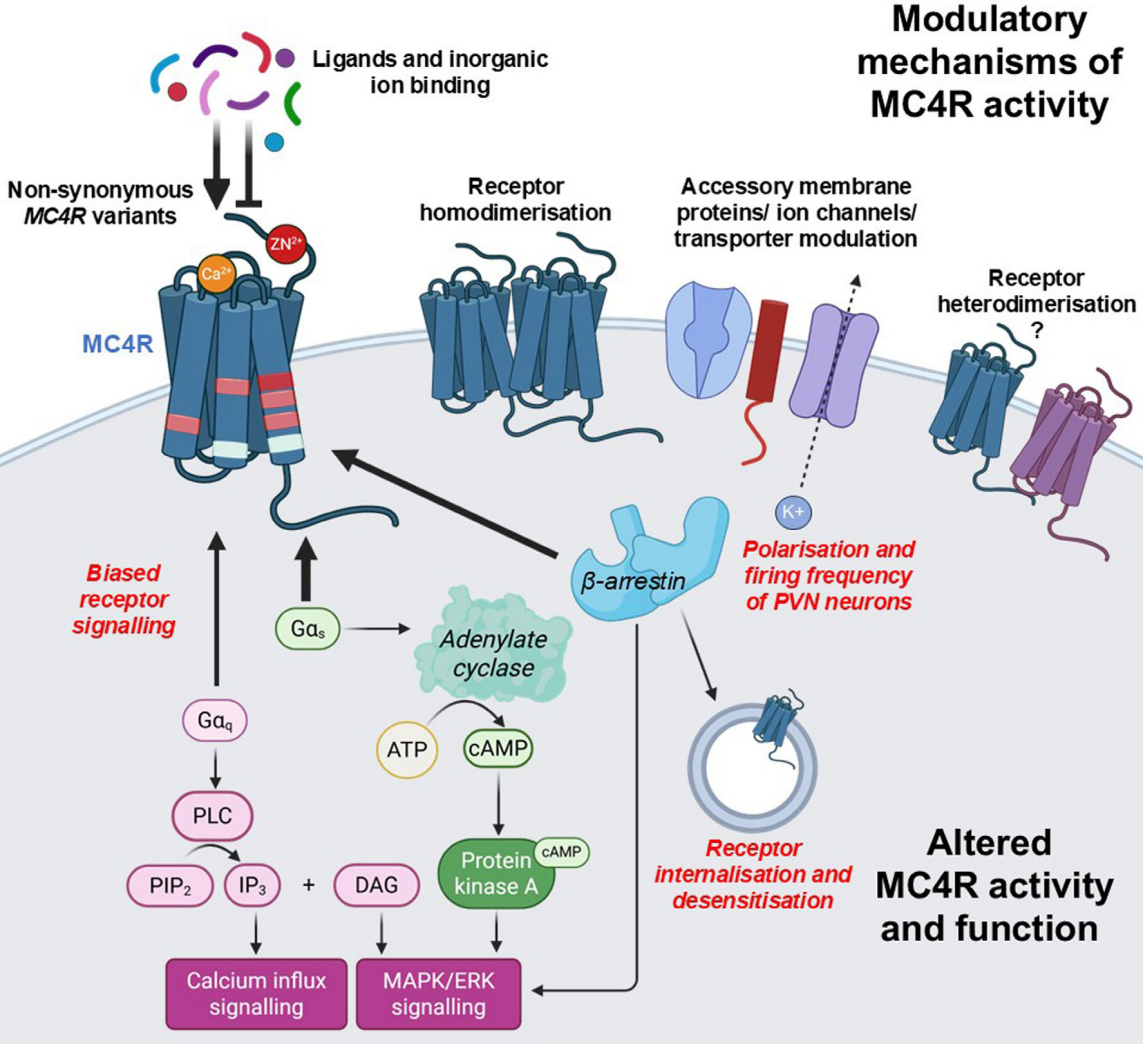
Regulators of MC4R signalling. Cartoon showing ways in which MC4R signalling is regulated. MC4R has been described to couple to all four G protein pathways but primarily acts upon the G_s_‐adenylate cyclase pathway to increase cAMP and can also utilise the G_q/11_‐phospholipase C (PLC) pathway to hydrolyse phosphatidylinositol 4,5‐bisphosphate (PIP_2_) into diacylglycerol (DAG) and inositol 1,4,5‐trisphosphate (IP_3_). Binding of IP_3_ to its receptor at the ER releases Ca^2+^ into the cytosol and DAG activates MAPK signalling. The activation of protein kinase A by cAMP and β‐arrestin can also enhance MAPK signalling.

GPCRs signal by four G protein signalling pathways: G_s_ that activates adenylate cyclase to increase cAMP, G_i/o_ that inhibits adenylate cyclase, G_q/11_ that stimulates phospholipase C to mobilise calcium from intracellular stores and G_12/13_ that activates RhoA to mediate cytoskeletal rearrangements. GPCR desensitisation occurs upon recruitment of β‐arrestin and internalisation by clathrin‐mediated endocytosis. The extracellular signal‐regulated kinase‐1/2 (ERK1/2) pathway can be activated downstream of several G protein or β‐arrestin pathways. MC4R has been reported to couple to all four G protein signalling pathways in cells,^4^, ^5^, ^6^ although the G_s_‐cAMP signalling pathway is best characterised and is impaired by MC4R mutations^1^ (Figure 1). In mice with a G_s_‐coupled DREADD (designer receptors exclusively activated by designer drugs) in MC4R‐expressing cells, chemogenetic activation significantly increased energy expenditure and locomotor activity, although a high dose of deschloroclozapine (a high affinity, selective DREADD agonist) was needed to suppress food intake.^7^ Mice with a global knockout of Gα_s_ had increased food intake, reduced energy expenditure, reduced heart rate with normal blood pressure and impaired insulin sensitivity and cold‐induced thermogenesis.^8^ In mice with PVN‐specific Gα_s_ deficiency, heterozygote mice developed obesity and had reduced energy expenditure,^9^ reduced blood pressure and heart rate, although cold‐induced brown adipose tissue stimulation and glucose metabolism were not changed.^9^ Brain‐specific deletion of the maternal Gα_s_ allele led to severe obesity with no effect on food intake or body length.^10^ These maternal Gα_s_ allele‐driven effects are due to *GNAS*, the gene encoding Gα_s_ being subject to genetic imprinting,^10^ which explains why only a subset of patients with Albright hereditary osteodystrophy caused by heterozygous loss‐of‐function Gα_s_ mutations develop obesity.^11^ Mutations in the *GNAS* gene that encodes Gα_s_ have also been identified in severe childhood‐onset obesity without characteristics of pseudohypoparathyroidism.^12^ Collected, the evidence indicates that Gα_s_ signalling is particularly important for the metabolic and cardiovascular effects of MC4R.

While most human obesity‐associated MC4R mutations do impair G_s_ signalling, either by reducing receptor cell surface expression or Gα_s_ coupling, approximately 25% of coding mutations associated with obesity do not affect this signalling pathway but instead reduce G_q/11_ signalling, phosphorylated ERK1/2 responses, β‐arrestin recruitment, receptor oligomerisation or receptor trafficking.^2^, ^12^ Mouse models have also indicated that MC4R‐mediated G_q/11_ signalling may have physiologically distinct functions from G_s_ signalling.^13^, ^14^, ^15^ Mice with hypothalamic‐depletion of Gα_q/11_ developed hyperphagia, severe obesity and high serum leptin and cholesterol, but had no changes in blood pressure or glucose metabolism.^13^, ^14^, ^15^ Moreover, the ability of an MC4R agonist to inhibit food intake was lost in mice lacking Gα_q/11_ in the PVN, but not in mice with deletion of Gα_s_.^14^

Combined, these studies suggest MC4R‐G_s_ signalling regulates energy expenditure, cardiovascular function, and glucose intolerance, while MC4R‐G_q/11_ signalling mediates changes in food intake, cholesterol and body length. The synthetic agonist, setmelanotide, more potently activates G_q/11_ signalling than α‐MSH. The ability of setmelanotide to control appetite with fewer side effects than previous generations of MC4R pharmacological agonists has been attributed to this biased signalling.^3^

A subset of human MC4R variants was associated with significantly lower BMI and lower odds of obesity, type‐2 diabetes and coronary artery disease.^16^ These protective variants have a signalling bias towards β‐arrestin recruitment and increased mitogen‐activated protein kinase signalling, which may arise from endosomal membranes.^2^ However, such signalling from intracellular locations will require further investigation as the most frequent gain‐of‐function variant, V103I, decreased agonist‐induced internalisation,^16^ which would be inconsistent with endosomal signalling, but could indicate prolonged signalling from plasma membranes driven by β‐arrestin scaffolding of signalling components.^17^

**FIGURE 2:**
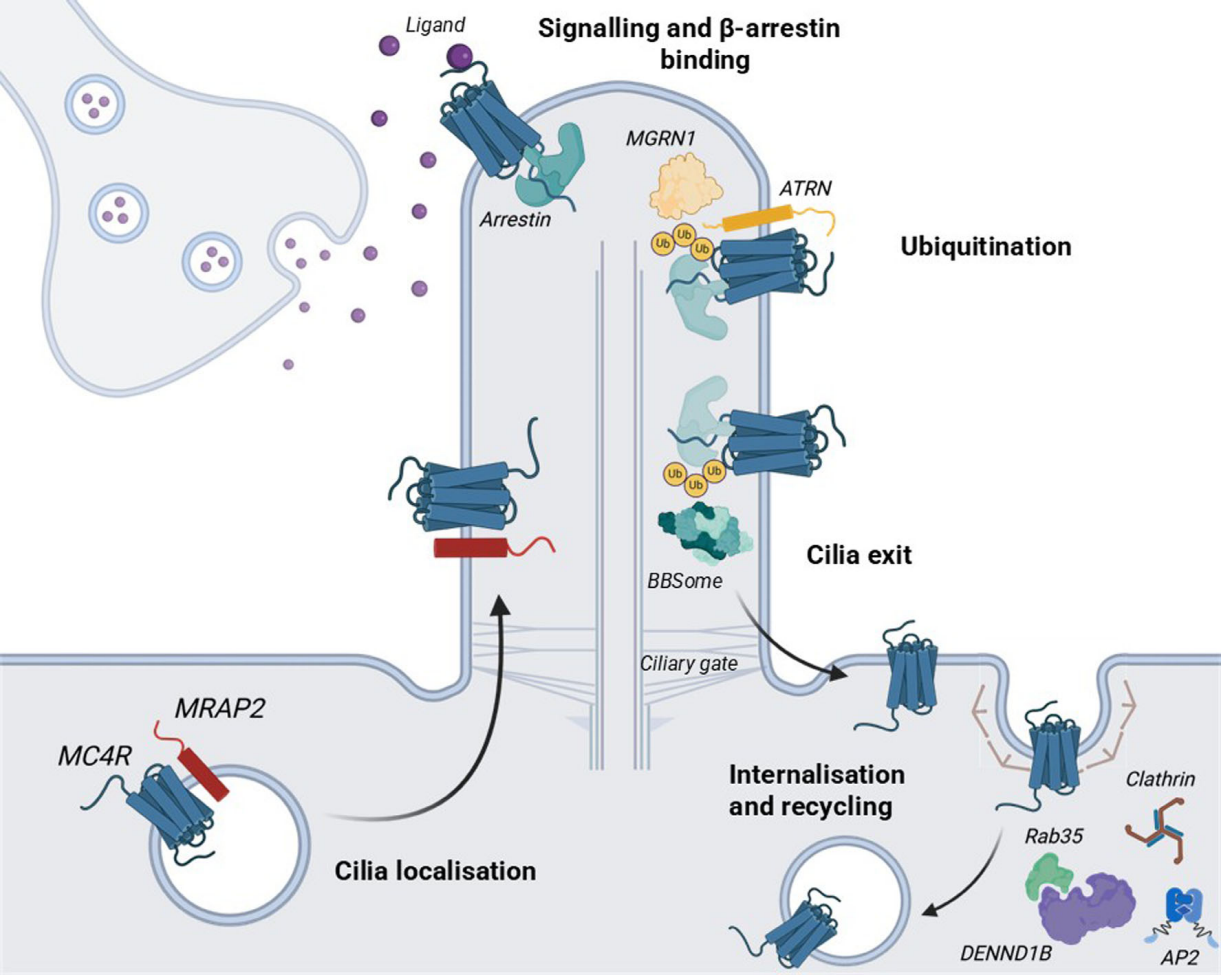
Regulators of MC4R trafficking. In neurons, MC4R localisation at primary cilia is crucial for its function. In addition to its role in modulating MC4R signalling, MRAP2 is required for MC4R ciliary localisation. MC4R desensitisation and removal from cilia is regulated by β‐arrestin and ubiquitination, and the latter pathway may involve MGRN1‐ATRN. DENND1B has been shown to regulate MC4R internalisation most likely by a clathrin and AP2‐dependent pathway.

Ciliary localisation of MC4R is important for receptor neuronal signalling (Figure 2). MC4R colocalises with adenylate cyclase type‐3 (ADCY3, itself mutated in monogenic obesity^18^) at the primary cilia of hypothalamic neurons, and a subset of human obesity‐associated MC4R mutations located in the third intracellular loop (ICL3) of the receptor impair ciliary localisation.^19^ Mice with deletion of functional cilia in MC4R‐expressing cells had severe obesity, increased fat and lean mass, increased body length and food intake, similar to global MC4R knockout mice.^20^ These mice no longer responded to MC4R agonists, suggesting that cilia are required for the anorexigenic functions of MC4R. Whether MC4R can also couple to G_q/11_ signalling pathways at cilia remains to be investigated. Cilia that express MC4R also shorten with age, which is associated with decreased energy expenditure and brown fat thermogenesis, leptin resistance, increased appetite and development of obesity.^21^ Despite this requirement for MC4R at primary cilia, MC4R ciliary expression in the adult brain is low under ad libitum fed conditions. This apparent paradox has been partially explained by a continuous depletion of MC4R from cilia, driven by β‐arrestin‐dependent ubiquitination.^22^ The authors suggest constitutive activation of MC4R drives β‐arrestin recruitment, ubiquitination and recognition by the BBsome and removal from cilia. However, previous studies have shown β‐arrestin recruitment is minimal under basal conditions,^4^, ^23^ and further evidence is needed from other studies to verify whether constitutive β‐arrestin recruitment drives the removal of MC4R from cilia. The inverse agonist AgRP, which is known to suppress MC4R constitutive basal activity, was able to impair ciliary exit.^22^ Combined, these studies suggest MC4R localisation at primary cilia is important for receptor function, but constitutive removal from cilia is required to regulate this signalling and may protect organisms from chronic starvation signals.

## 3 LIGAND AND DIVALENT ION COFACTORS MODULATE MC4R SIGNALLING

The first structures of MC4R showed calcium is a critical cofactor for α‐MSH and NDP‐MSH agonist binding and efficacy.^24^, ^25^, ^26^ Ca^2+^ binding is coordinated by three conserved negatively charged residues (E^2.60^, D^3.25^, and D^3.29^) within MC4R and residues of the ligands.^25^, ^26^ The calcium ion forms a link between the ligands and TM2 and TM3 of MC4R.^26^ Ca^2+^ improves the potency of setmelanotide but has minimal effects on AgRP or other MC4R antagonists.^24^, ^25^ When these calcium‐coordinating residues are mutated, they decrease MC4R efficacy for α‐MSH and setmelanotide under physiological Ca^2+^ concentrations and cause obesity.^24^ Setmelanotide forms unique interactions with Ca^2+^ and TM3 residues in MC4R that favour receptor activation when compared to NDP‐α‐MSH.^26^ Whether Ca^2+^ binding is important for constitutive activation of the receptor is inconclusive; some studies identify Ca^2+^ in the absence of ligand, suggested to prime the receptor for activation,^24^ while others do not.^27^ Other divalent ions did not have similar effects to Ca^2+^ on receptor activation.^24^, ^25^ However, structural and biochemical studies suggest that Zn^2+^ may modulate MC4R constitutive activity.^27^, ^28^

## 4 MC4R OLIGOMERISATION

MC4R has been shown to homooligomerise in transfected HEK293 cells by BRET,^2^ FRET,^29^ Fluorescence Cross‐Correlation Spectroscopy (FCCS)^30^ and single‐molecule microscopy.^31^ The mechanism by which MC4R receptors associate is not yet known. However, this does not involve di‐sulphide bonds that are essential for homodimerisation of class C GPCRs, as mutagenesis of cysteine residues in the MC4R extracellular region, which would be required for di‐sulphide bonds, had no effect on dimerisation.^32^ Mutagenesis studies that prevent MC4R oligomerisation led to increased receptor signalling in some studies and it was hypothesized that dissociation of MC4R oligomers to monomers may increase signalling capacity.^33^ However, multiple MC4R mutants that are associated with obesity and therefore confer reduced receptor activity have also been shown to disrupt MC4R homodimerisation.^2^ These mutations affect other aspects of MC4R signalling (e.g., Gα_s_ coupling, β‐arrestin recruitment) and it is therefore difficult to correlate the reduced homodimerisation with decreased MC4R activity.^2^ Previous studies showed that MC4R mutant proteins may heterodimerise with wild‐type MC4R and induce a dominant‐negative effect.^29^ None of these studies have investigated endogenous MC4R protein, something that is difficult with currently available techniques; however, the elucidation of a MC4R homodimer structure would advance understanding of receptor oligomerisation and could emphasise its importance in the physiological state.

Interaction between the MC4R and several other GPCRs has been reported in cell studies. MC4R can immunoprecipitate with 11 GPCRs^34^ and interaction between MC4R and GPR7 has been shown by FRET.^35^ However, effects on signalling were only investigated in one study^34^ with overexpression of putative GPCR partners (1:3, 1:7 MC4R:GPCR). The physiological significance of such interactions has not been investigated.

## 5 MODULATION BY OTHER TRANSMEMBRANE PROTEINS

### 5.1 Inwardly‐rectifying potassium (Kir) channels

An MC4R‐driven G protein‐independent signalling pathway that involves inwardly‐rectifying potassium channel 1.7 (Kir1.7) has been described to modulate energy homeostasis. This pathway was identified following observations in mouse hypothalamic brain slices in which α‐MSH and AgRP‐driven changes in action potential firing in PVN neurons (activation and hyperpolarisation, respectively) were shown to be resistant to the inhibition of G_s_ signalling.^36^ Instead, the regulation of firing activity was mediated by α‐MSH‐induced closure of Kir7.1, while AgRP opens the Kir7.1 channel.^36^ Modified MC4R that could not interact with Gα_s_ was still able to couple to Kir7.1.^25^ These Kir7.1‐driven effects are distinct from the cAMP‐PKA‐mediated K_ATP_ channel activation that has been described to contribute to MC4R‐induced hyperpolarisation in the brainstem.^37^ Additionally, MC4R‐mediated peptide YY secretion in peripheral L cells did not require Kir7.1,^38^ indicating that some MC4R signalling is Kir7.1‐independent.

Global deletion of the Kir7.1 gene, *Kcnj13*, resulted in early postnatal lethality.^38^ Therefore, the effects of Kir7.1 have been assessed in mice with conditional knockout of *Kcnj13* from MC4R expressing cells (*Kcnj13ΔMC4R* ^*Cre*^). Hypothalamic slices from these mice have defective α‐MSH‐induced depolarisation of MC4R PVN neurons. Upon infusion of a potent α‐MSH analogue, LY2112688, these conditional knockout mice had reduced fasting‐induced refeeding responses.^38^ Findings were similar in mice with lentiviral *Kcnj13* shRNA knockdown in the PVN. *Kcnj13ΔMC4R* ^*Cre*^ mice had age‐related increased body weight, increased length and impaired glucose metabolism, although these changes were not observed in younger mice.^38^ When fed a high‐fat diet, *Kcnj13ΔMC4R* ^*Cre*^ mice had increased body weight and fat mass, but no change in food intake.^38^

Those findings could indicate that Kir7.1 has a more nuanced effect on MC4R than suggested in hypothalamic slices or that there are developmental effects that may not be present if Kir7.1 was deleted in adults. This question has been partially addressed by studies with an inhibitor of Kir7.1, ML418. This compound potently inhibits conductance through the Kir7.1 channel and prevents the α‐MSH‐induced depolarisation of MC4R neurons. In wild‐type mice, ML418 reduced 24‐h food intake and induced weight loss in a dose‐dependent manner.^39^ This provides evidence that in conditional knockouts of *Kcnj13* there may be compensatory mechanisms during development. Coupling of Kir7.1 to MC4R is important for mediating the effects of several drugs on eating and weight gain. These include the effects of cannabinoids on MC4R‐expressing neurons^40^ and antipsychotics that increase hyperphagia and weight gain by promoting the Kir7.1 open state and inhibiting the activity of MC4R‐expressing neurons.^41^

Whether MC4R and Kir7.1 form a direct complex is still inconclusive. Studies of MC4R and Kir7.1 artificially linked with three different tethers demonstrate no effect on cAMP EC_50_ values but show impaired maximal activity. Cryo‐EM reconstructions from a small number of particles with one of these linkers and AlphaFold predictions suggested the agonist‐bound MC4R undergoes a tilted conformation allowing G protein binding and disengagement from Kir7.1, which subsequently closes the channel.^39^ Interactions are predicted between MC4R TM5 at the extracellular interface and ICL2 and ICL3 with the intracellular Kir7.1 interface that helps maintain the channel open conformation.^39^ Other Kir channels have similar tetrameric structures with conserved selectivity filter regions required for conducting K^+^ across the channel. While modulation of MC4R is not a shared mechanism for all Kir channels,^36^ MC4R has recently been shown to negatively regulate the expression of Kir2.1, and this was suggested to affect insulin sensitivity.^42^ Upon MC4R overexpression, Kir2.1 expression is reduced in mouse hypothalamic GT1‐7 cells and, reciprocally, Kir2.1 levels are elevated in MC4R‐deficient mice.^42^ While co‐immunoprecipitation assays suggested that MC4R and Kir2.1 may interact, how this results in changes in the expression of Kir2.1 or how MC4R signalling is modulated was not investigated.^42^

### 5.2 Melanocortin‐2 receptor accessory protein 2 (MRAP2)

Melanocortin‐2 receptor accessory protein 2 (MRAP2) is a single‐pass transmembrane protein that is highly expressed in the brain^43^ and has been shown in multiple studies to facilitate MC4R signalling.^30^, ^44^, ^45^, ^46^ It was originally identified as a homolog to MRAP1, a transmembrane protein that is essential for the cell surface expression and signalling of the melanocortin‐2 receptor (MC2R) that regulates adrenal development.^43^ While both MRAP1 and MRAP2 co‐immunoprecipitate with MC4R when overexpressed in cells,^43^ MC4R does not have a major role in adrenal cells and MRAP1 modulation of MC4R will not be discussed further. In the initial study that identified MRAP2 as a possible MC4R binding protein, overexpression of MRAP2 reduced MC4R cell surface expression and NDP‐MSH‐mediated cAMP responses in CHO cells.^43^ However, subsequent in vitro and Zebrafish studies demonstrated that MRAP2 enhanced MC4R‐mediated cAMP responses.^45^, ^46^ MRAP2 has also been shown to enhance MC4R‐driven G_q/11_ signalling.^30^, ^44^

Global and brain‐specific *Mrap2*‐null mice develop obesity and heterozygous mice are significantly heavier than wild‐type animals,^45^ analogous to *Mc4r*‐knockout mice, providing further evidence that MRAP2 regulates MC4R activity. Moreover, rare MRAP2 variants that inactivate MC4R function are associated with obesity and overweight in human populations.^44^, ^45^ However, MRAP2 also regulates other GPCRs including the ghrelin receptor^47^ and MC3R,^48^ which has partially explained why mice with *Mrap2* and *Mc4r* deleted simultaneously are not as severely affected as *Mc4r*‐null mice,^45^ and why humans with loss‐of‐function *MRAP2* variants may have additional phenotypes when compared to individuals with *MC4R* mutations alone.^49^

Further investigation of mice with *Mrap2* deleted from MC4R neurons showed that the accessory protein is important for cardiovascular autonomic regulation.^50^ MC4R agonist‐driven increases in sympathetic nerve activity of renal and brown adipose tissue and hepatic vagal nerve activity were attenuated in mice with *Mrap2* depleted from MC4R neurons. This was attributed to the projection of MC4R neurons from the brain to the kidneys and brown adipose tissue.^50^ This was associated with a significant decrease in heart rate. However, these mice did not have hypertension, suggesting this phenotype reported in humans with inactivating *MRAP2* variants is likely due to effects on receptors other than MC4R.^50^

Early studies suggested that MRAP2 may form homodimers at the cell surface^43^ and both parallel and antiparallel dimers have been described.^43^, ^51^ However, others have been unable to replicate these antiparallel dimers.^22^, ^52^ Single‐molecule pull‐down (SiMPull) and total internal reflection fluorescence microscopy have demonstrated that MRAP2 can form dimers, but that the majority of MRAP2 at cell surfaces is monomeric.^48^ Moreover, structural homology models of MRAP2 in complex with MC4R suggest a 1‐to‐1 stoichiometry^30^, ^44^ and SiMPull with photobleaching step analysis confirmed that monomeric MRAP2 interacts with monomeric MC4R at the cell surface.^31^ The predicted interaction interface involves MC4R TM5 and TM6 and the MRAP2 transmembrane region; mutation of MRAP2 residues within this interaction interface reduces MC4R activity.^44^ Notably, MRAP2 has been overexpressed at 3–8× the concentration of MC4R in cell studies.^43^, ^45^, ^46^, ^49^ However, others showed that MRAP2 enhanced MC4R G_s_ and G_q_‐mediated activity when plasmids were expressed at a 1‐to‐1 ratio,^30^, ^44^ suggesting that overexpression of MRAP2 is not required for the functional effects on MC4R.

Several mechanisms have been described to explain how MRAP2 affects the activity of MC4R. MRAP2 decreases β‐arrestin‐2 recruitment to MC4R and reduces MC4R constitutive and ligand‐mediated internalisation,^30^, ^31^ resulting in a more active receptor at the cell surface. The MRAP2 cytoplasmic region is critical for facilitating MC4R signalling and structural studies suggest it may prevent β‐arrestin‐2 from accessing the GPCR.^31^, ^44^ Obesity‐associated MRAP2 variants affect residues that are located intracellularly and are predicted to affect G protein and/or β‐arrestin binding.^44^ However, the prevention of β‐arrestin‐2 recruitment alone is insufficient to explain MRAP2 effects on MC4R activity, as the accessory protein can still promote G_s_ recruitment in β‐arrestin‐1/2 knockout cells.^30^ One additional mechanism could be the alteration of MC4R oligomerisation by MRAP2 that favours a monomeric state.^30^, ^31^ Additionally, MRAP2 is required for MC4R enrichment at cilia,^22^, ^53^ although the residues involved in this process and the mechanism are still to be elucidated.

### 5.3 Transmembrane proteins with limited evidence

MC4R has been described to interact with two other transmembrane proteins in a single study to date. The opsin 3 receptor (OPN3) is a GPCR that is co‐expressed with MC4R in the PVN of the mouse brain.^54^ OPN3 is coupled to G_i/o_ signalling pathways that reduce cAMP levels. Knockdown of OPN3 in hypothalamic GT1‐7 cells enhanced cAMP responses evoked by the MC4R agonist MTII compared to control cells. Preincubation of cells with pertussis toxin to block G_i/o_ activity prevented the OPN3‐dependent suppression of cAMP^54^ suggesting OPN3 negatively regulates MC4R‐mediated signalling. In PVN brain slices from mice with conditional deletion of *Opn3* from *Mc4r*‐expressing neurons there was an increase in spontaneous firing rate and a reduced number of open Kir7.1 channels suggesting OPN3 may potentiate Kir7.1 activity.^54^ OPN3 constitutive activity modulated the basal activity of Kir7.1 channels in a Gαi/o‐dependent manner but this was independent of MC4R. OPN3 immunoprecipitated with MC4R and Kir7.1 suggesting the three proteins may form a complex.

Mice with conditional deletion of *Opn3* from *Mc4r*‐expressing neurons had reduced food consumption and locomotor activity.^54^ OPN3 constitutive activity therefore negatively regulates MC4R cAMP signalling and potentiates Kir7.1. OPN3 has previously been shown to form a complex with MC1R and similarly reduces its activity,^55^ although it does not affect other GPCRs tested, suggesting this may be a specific effect on melanocortin receptors. Future studies will be needed to determine the endogenous ligand for OPN3 in hypothalamic cells and whether OPN3‐mediated regulation of MC4R has physiological functions in other species including humans.

Monocarboxylate transporter 8 (MCT8) has previously been characterised as a thyroid hormone transporter,^56^ but is also co‐expressed with MC4R in hypothalamic neurons.^57^ NanoBRET assays suggested that MC4R and MCT8 may interact in HEK293 cells.^57^ MCT8 did not affect MC4R‐mediated cAMP signalling but reduced MC4R‐mediated G_q_ signalling.^57^ MCT8 expression did not alter total or surface expression of either protein. However, the physiological effect of this interaction and the mechanism by which MCT8 modulates G_q_‐ but not G_s_‐coupling of MC4R still remain to be explored.

## 6 REGULATORS THAT PRIMARILY AFFECT MC4R CELL SURFACE EXPRESSION OR TRAFFICKING

The expression of MC4R at cell surfaces is critical for its engagement with G proteins and downstream signalling pathways. The majority (78% in one study,^2^ 91% in another^58^) of human obesity‐associated MC4R mutations impair receptor trafficking to the cell surface due to protein misfolding and consequent ER retention^2^, ^58^ and pharmacological rescue by chaperones have been investigated as potential treatment options in some cases.^59^, ^60^ Variants that protect from obesity have reduced agonist‐induced internalisation and both protective and obesity‐associated MC4R variants modulate β‐arrestin recruitment.^16^ Several proteins have been described that also primarily regulate MC4R cell surface expression or internalisation, and although many have only been described in a single study, cumulative evidence of effects on trafficking suggest these proteins may collectively contribute to MC4R function. We will discuss several of these proteins and how they are thought to regulate MC4R expression.

### 6.1 DENN domain‐containing protein 1B

Variants in DENN domain‐containing protein 1B (*DENND1B*) have recently been associated with both canine and human obesity phenotypes using unbiased genomic approaches.^61^ Firstly, single nucleotide polymorphisms within an intronic region of *DENND1B* were the most significant association with canine adiposity in a genome wide association study (GWAS) of 241 Labrador retrievers. A region of association was also identified in a large human GWAS for BMI and *DENND1B* assigned as the most likely causal gene of the association. In addition, rare damaging variants in *DENND1B* were associated with BMI in the UK Biobank study.^61^ DENN domain containing proteins act as guanine nucleotide exchange factors (GEF) that mediate the exchange of GDP for GTP on small Rab GTPases, which are molecular switches for a variety of cellular processes. DENND1B is known to have GEF activity for Rab35 and Rab15.^62^ DENND1B, Rab35 and Rab15 contribute to clathrin‐mediated endocytosis and endocytic recycling with DENND1B directly interacting with clathrin and AP‐2, the latter at the same position as β‐arrestin.^63^ Rab35 is also known to regulate receptor levels within the cilia.^64^

Co‐expression of *DENND1B* and *MC4R* in human hypothalamic cells suggested DENND1B could regulate MC4R trafficking.^61^ In vitro, DENND1B expression directly influenced MC4R signalling and trafficking in HEK293 cells, with DENND1B overexpression reducing cAMP signalling and increasing agonist‐induced MC4R internalisation without altering MC4R surface expression.^61^ Inversely, *DENND1B* knockdown increased cAMP signalling and decreased MC4R internalisation.^61^ Direct protein interactions were not investigated between DENND1B and MC4R, and the mechanism for DENND1B‐dependent regulation of MC4R remains unclear. However, GEFs are thought to be crucial for the spatial and temporal localisation of Rab GTPases,^62^, ^65^ and DENND1B is required for Rab35 localisation at the cilium.^66^ The role of DENND1B and Rab35 in MC4R function at the cilia is currently unexplored and studies in ciliated cells may be required to elucidate the specific mechanisms of MC4R regulation.

### 6.2 ER chaperones

ER stress and activation of the unfolded protein response (UPR) augment obesity in animals on a high‐fat diet by several mechanisms including development of leptin resistance,^67^ while treatment of obese mice with chemical chaperones that prevent ER stress can increase insulin sensitivity.^68^ ER chaperones have been shown to regulate MC4R activity including heat shock cognate 70 (Hsc70) and the Glucose‐regulated protein 78 (GRP78, also known by its gene name *Hspa5* [Heat shock protein family A member 5]).

Hsc70 immunoprecipitated with MC4R and enhanced the surface expression and signalling of mutant receptors. However, while Hsc70 increased MC4R total protein expression, it had no effect on wild‐type surface expression.^69^ Moreover, overexpression of Hsc70 did not alter the diffusional mobility of WT MC4R in membranes.^69^ Therefore, Hsc70 has a role in rescuing mutant MC4R from ER retention but may not have a major role in normal MC4R function.

GRP78 was identified as an MC4R regulatory protein in GST‐pulldown assays in mouse hypothalamic extracts using the recombinantly expressed ICL3 of MC4R as bait.^70^ GRP78 is primarily expressed at the ER where it performs its functions in the unfolded protein response (UPR). Overexpression of GRP78 in the ventromedial hypothalamus of rats alleviates ER stress and obesity^71^ and variants in human *HSPA5* are associated with BMI, body weight and lean body mass.^72^, ^73^ Mechanistic studies to understand how GRP78 may regulate MC4R activity showed that agonist‐stimulated internalisation of MC4R increased colocalization between GRP78 and MC4R at intracellular sites, while knockdown of *GRP78* in cells reduced MC4R internalisation, CRE luciferase activity and increased UPR activation upon ER stress.^70^ Specific inhibition of GRP78 with HA15 reduced MC4R and G_s_ expression as well as cAMP‐PKA molecules in porcine embryos, although it is unclear if the changes in MC4R levels directly affect cAMP levels.^74^ In mice, injection of the PVN with *GRP78*‐targeting lentivirus had no significant effect on food intake, body weight, oxygen consumption or locomotor activity on normal or high fat diet when compared to lentivirus control injected mice.^70^ Therefore, the physiological relevance of GRP78 regulation of MC4R is still to be determined.

### 6.3 Mahogunin ring finger 1 and Attractin‐like 1

Mahogunin ring finger 1 (MGRN1) is a membrane‐tethered E3 ligase with known roles in ubiquitination and endosome‐to‐lysosome trafficking.^75^ MGRN1 has been implicated in melanocortin receptor signalling as mutations in murine *Mgrn1* and its binding partner Attractin (*Atrn*) produce a lean phenotype by suppressing the effect of the inverse agonist agouti‐signalling protein (ASIP) on MC4R and MC1R.^76^, ^77^ It was functionally determined that MGRN1 or *Atrn* loss‐of‐function prevents ASIP‐dependent degradation of MC4R and promotes recycling of MC4R to the cell surface.^76^ It was initially speculated that MGRN1 regulation of MC4R was independent of traditional E3 ubiquitin ligase pathways (ubiquitination and degradation).^78^ However, subsequent evidence suggested MGRN1 ubiquitination does occur but only when in complex with the transmembrane proteins ATRN or ATRNL1 (Attractin‐like 1).^78^, ^79^ Further reiterating the role of ubiquitination in MC4R ciliary exit, double knockout of E3 ligases *Mgrn1* and ring‐finger 157 (*Rnf157*, its vertebrate‐specific analog) increased total MC4R expression levels and ciliary localisation in cells.^79^ A possible interaction between MC4R and MGRN1 was identified by co‐immunoprecipitation^79^ and was suggested to suppress MC4R signalling by directly competing with Gα_s_ to bind to MC4R.^80^ This was supported by studies in which overexpression of Gα_s_ rescued MGRN1‐dependent reductions in cAMP signalling.^78^

More recently, ATRNL1 has been shown to interact with MC4R,^81^ confirming initial yeast‐two‐hybrid studies over 20 years ago.^82^ Similarly to MRAP2, ATRNL1 enhances MC4R‐mediated cAMP signaling by reducing β‐arrestin recruitment.^81^ Deletion of *Atrnl1* from the PVN in mice increased food intake and weight gain without affecting energy expenditure. This could not be rescued by MC4R agonist treatment, suggesting ATRNL1 has a functional role in the MC4R pathway.^81^ Rare variants in the *ATRNL1* gene that impaired MC4R signaling were identified in children with early‐onset obesity; however genetic association studies in larger cohorts are needed to assess whether *ATRNL1* variants contribute to the risk of obesity.^81^

### 6.4 C2CD5

The calcium‐ and lipid‐binding C2 domain (C2CD5) protein was recently identified to regulate MC4R trafficking.^83^ C2CD5 is expressed in MC4R‐positive cells of the PVN.^83^ Knockout of murine *C2CD5* caused obesity with reduced energy expenditure and these mice were resistant to MC4R agonist treatment when ligand was directly injected into the PVN.^83^, ^84^ Additionally, hypothalamic C2CD5 expression in mice is reduced after high fat diet feeding.

Previously, the only documented role of C2CD5 was in GLUT4 translocation to the adipocyte plasma membrane after insulin stimulation.^85^ Therefore, C2CD5 was hypothesised to regulate MC4R endocytosis. Overexpression of C2CD5 in cells enhanced MC4R internalisation but did not affect recycling.^83^ However, the loss of the C2 domain that is required for C2CD5 to perform its trafficking function had no effect on cAMP signalling. Further study of C2CD5 on MC4R activity is required, not least because its effects on MC4R signalling have not been investigated. Moreover, although evidence supports a C2CD5 regulatory role in MC4R trafficking, the underlying mechanism remains unclear.

## 7 NOVEL ENDOGENOUS AGONISTS (LIPOCALIN‐2, NESFATIN‐1, Β‐DEFENSINS)

Recent discoveries of MC4R endogenous ligands outside the POMC or AgRP families introduce further complexity to MC4R pharmacology in vivo. While these novel ligands have shown MC4R binding and receptor signalling, additional characterisation is required to determine their distinct pharmacology and physiological relevance.

Lipocalin‐2 (LCN2) was originally described as an adipokine^86^ with anorexigenic effects in mice and primates,^87^, ^88^ and later identified in bone tissue with specific secretion from osteoblasts.^87^ Deletion of osteoblast‐derived LCN2 increased food intake in mice, intraperitoneal administration of LCN2 to wild‐type mice reduced food intake, fat mass and body weight, serum levels of LCN2 were elevated after refeeding wild‐type mice after overnight fasting, and serum levels of LCN2 inversely correlated with body weight in humans.^87^ LCN2 has been shown in independent studies to mediate appetite suppression in pancreatic cancer cachexia^89^ and exogenous treatment of mice with LCN2 can normalise feeding and body weight gain in response to selective serotonin reuptake inhibitor (SSRI) antidepressants.^90^ Moreover, LCN2 suppresses appetite in humans and monkeys.^88^ LCN2 mediates its effects by crossing the blood–brain barrier and binding with high affinity to hypothalamic MC4R.^87^, ^88^, ^89^ The anorexigenic effects of LCN2 are lost when MC4R is deleted from mice. In HEK293T and GT1‐7 hypothalamic cells LCN2 induced MC4R‐mediated cAMP activation, without activating ERK or tyrosine kinase phosphorylation.^87^ Further study is needed to characterise LCN2 signalling in more detail including investigating G protein recruitment to MC4R to verify the signalling bias suggested in these studies.

Nesfatin‐1 is an 82 amino acid cleavage product of nucleobindin‐2,^91^ and another putative endogenous ligand of MC4R. It is expressed in the PVN and peripheral tissues and reduces feeding and body weight in animals.^91^ Pre‐treatment of rats with the SHU9119 antagonist abolished nesfatin‐1‐induced satiety^91^ and nesfatin‐1 knockdown in the PVN of rats reduced *Mc4r* mRNA levels.^92^ Nesfatin‐1 is linked to brown adipose tissue thermogenesis by upregulating uncoupling protein‐1 via the melanocortin pathway.^93^ SHU9119 acutely inhibits the nesfatin‐1 thermogenic effect^94^ and nesfatin‐1 knockdown in rat PVN abolishes stress‐induced thermogenesis.^92^ Several studies have indicated that the nesfatin‐1 receptor is present on the mouse hypothalamus, that nesfatin‐1 influences CREB signalling in cells expressing MC4R and that these effects are abolished by SHU9119 treatment.^95^ MC4R on enteroendocrine L cells can also regulate hepatic glucose production in a pathway involving nesfatin‐1 and GLP‐1.^96^ These findings led to the exploration of whether nesfatin‐1 can bind to MC4R. The two proteins co‐immunoprecipitate in intestinal secretory tumor cells and complex formation was confirmed by BRET.^96^ Mutagenesis and molecular dynamics simulations identified a short sequence of nesfatin‐1 (the HFR domain) that may interact with the MC4R ligand binding site. Infusion of nesfatin‐1 with deletion of this sequence into the duodenum of rats abolished the effect of nesfatin‐1 on cell signalling and on hepatic glucose production.^96^ Such a direct effect of nesfatin‐1 on MC4R could also explain observations in cardiomyocytes where nesfatin‐1 has been reported to suppress L‐type Ca^2+^ channels through the MC4R‐PKC_θ_ pathway.^97^

β‐Defensins are peptides primarily secreted by epithelial skin tissue which can modulate MC4R activity. Initially linked to MC1R via canine β‐defensin 103, which confers black coat colour in dogs,^98^ the effect of human β‐defensin 3 (HBD3) on MC4R is still unresolved. In transfected HEK293 cells, HBD3 can activate cAMP and pERK1/2 signalling in the micromolar range.^99^, ^100^ In rodent pharmacological studies, HBD3 acts as a neutral melanocortin receptor antagonist, that blocks both agonist and inverse agonist binding, a mechanism which may be conferred by HBD3's unique ability to interact with MC4R using asymmetric clusters of positively charged patches that allows charge‐driven promiscuous binding.^101^ The centrally expressed BD1 also acts as a micromolar agonist for MC4R in transfected HEK293, although there was no significant effect of BD1, even at 100 μg doses, on body weight or food intake.^100^ As it is unknown whether β‐defensins are present in the micromolar range in humans or mice, it is unclear whether they have any physiological effect on MC4R.

## 8 CONCLUSION

Since the discovery of the MC4R and the elucidation of its critical function in appetite, there have been many important advances in understanding how other proteins and cofactors can influence its activity. Many of these pathways and interactions have only recently been uncovered and further investigation of them is likely to yield further insights into the regulation of MC4R signalling and trafficking and perhaps identify novel pharmacological targets.

## AUTHOR CONTRIBUTIONS

**Aqfan Jamaluddin:** Writing – review and editing; visualization. **Alyce McClellan:** Writing – review and editing; visualization. **Eleanor Raffan:** Writing – review and editing. **Caroline M. Gorvin:** Writing – review and editing.

## CONFLICT OF INTEREST STATEMENT

The authors declare no conflict of interest.

## Data Availability

Data sharing not applicable to this article as no datasets were generated or analysed during the current study.
